# The association between unemployment and treatment among adults with hemophilia

**DOI:** 10.1016/j.rpth.2024.102514

**Published:** 2024-07-14

**Authors:** Christian Qvigstad, Lars Q. Sørensen, Geir E. Tjønnfjord, Pål André Holme, Age-related DeVelopments ANd ComorbiditiEs in hemophilia Working Group, Ingrid Pabinger, Cedric Hermans, Roseline d’Oiron, Robert Klamroth, Johannes Oldenburg, Natascha Marquardt, Peter Staritz, Olga Katsarou, Uri Martinowitz, Aharon Lubetsky, Gili Kenet, Annarita Tagliaferri, Maria Elisa Mancuso, Roger Schutgens, Pål André HolmE, Jerzy Windyga, Irena Zupan, Victor Jimenez Yuste, Ramiro Nunez, Philippe de Moerloose, Erik Berntorp, Jan Astermark, Campbell Tait, Gerry Dolan

**Affiliations:** 1 Department of Haematology, Oslo University Hospital Rikshospitalet, Rikshospitalet, Norway; 2 Research Institute of Internal Medicine, Oslo University Hospital Rikshospitalet, Rikshospitalet, Norway; 3 Institute of Clinical Medicine, University of Oslo, Oslo, Norway; 4 Independent researcher, Oslo, Norway

**Keywords:** hemophilia A, hemophilia B, treatment, unemployment, workforce

## Abstract

**Background:**

People with hemophilia often experience pain and suffer from comorbidities related to their bleeding disorder. Consequently, unemployment due to disability is prevalent among people with hemophilia.

**Objectives:**

To explore associations between unemployment due to disability and treatment while adjusting for known risk factors for unemployment.

**Methods:**

Collecting data from 20 hemophilia centers from 15 European countries, the Age-related DeVelopments ANd ComorbiditiEs in hemophilia study recruited 785 participants aged 40 years and over with hemophilia A or B. A comprehensive electronic case report form included items related to patient characteristics, demographic information, past and current treatment regimens, and medical history, including a lifelong history of comorbidities. Baseline data from the Age-related DeVelopments ANd ComorbiditiEs in hemophilia study was analyzed using descriptive statistics and logistic regression models.

**Results:**

Employment status was available for 756 of 785 participants aged 40 to 88 years (median, 53 years). We used regression analysis to compare people with hemophilia who were fully employed with those who were unemployed due to disability. This analysis included 424 participants. Using multivariable logistic regression, we found that age (odds ratio [OR], 1.07; *P* < .01), severe hemophilia (OR, 10.81; *P* < .01), current smoker (OR, 2.53; *P* < .01), and psychiatric disorder (OR, 4.18; *P* = .02) were associated with increased odds of unemployment due to disability. In contrast, prophylactic treatment (OR, 0.44; *P* = .01) was associated with decreased odds.

**Conclusion:**

Our analysis suggests that by maintaining factor levels above a critical threshold (3%-5%), prophylactic treatment for people with hemophilia could help avoid unemployment due to disability. While prophylaxis is more costly and can be burdensome, the benefits to material well-being and quality of life could be substantial.

## Essentials

• Unemployment is more prevalent for adults with hemophilia than for adults without disabilities.
• The study recruited 785 people with hemophilia A or B from 15 European countries.
• Prophylactic replacement therapy was negatively associated with unemployment due to disability.
• We recommend prophylactic rather than on-demand treatment to always ensure adequate factor levels.

## 1 Introduction

Hemophilia is an inherited deficiency or dysfunction of specific clotting proteins associated with bleeding upon hemostatic challenges and recurrent and spontaneous bleeding. Hemophilia A is caused by a lack of clotting factor (F)VIII, while hemophilia B is caused by a lack of clotting FIX. The severity of bleeding in hemophilia varies from mild to severe, and the degree of factor deficiency often corresponds to the severity of bleeding. Severe hemophilia is characterized by spontaneous bleeding into joints and muscles. Recurrent bleeds in the same joint, a target joint, can lead to chronic damage known as hemophilic arthropathy and potentially severe disability, if not treated properly [1]. Replacement therapy with clotting factor concentrates is used to treat bleeding episodes. This can be done on demand, which is episodic replacement therapy in response to an acute bleed, or through prophylaxis, which is regular replacement therapy to prevent bleeding [2]. Although new treatment options are emerging, factor replacement therapy remains crucial, and prophylaxis is considered the gold standard [3]. While factor replacement has significantly reduced joint bleeding, many individuals born before the availability of clotting factor concentrates or early regular prophylaxis suffer the crippling musculoskeletal effects of hemophilic arthropathy. Chronic pain and joint damage can lead to a downward spiral of limited mobility, osteoporosis, overweight or obesity, and declining overall health [[4], [5], [6]]. This disability further impacts people with hemophilia as research shows those with mobility limitations are less likely to be employed [7].

Previous research has reported that hemophilia had a negative impact on employment [[8], [9], [10]]. This is corroborated by a global report from the World Federation of Hemophilia which found that “hemophilia affected the employment status of 18% of people with hemophilia, forcing them into part-time employment, long-term sick leave, unemployment or retirement” [11]. These studies did not report the risk factors most strongly associated with unemployment or whether any forms of treatment kept people with hemophilia in the workforce. The aim of the current study was to compare unemployment prevalence among people with hemophilia with Organisation for Economic Co-operation and Development (OECD) averages for the nondisabled general population. Disability is defined by OECD as people who 1) declared that they suffer from any chronic illness or condition and 2) have moderate to severe activity limitation due to health problems [12]. Thus, all people with hemophilia can reasonably be said to suffer from a disability. Moreover, this study was conducted to better understand the association between employment and hemophilia treatment after adjusting for clinically relevant variables that may also affect employment status for people with hemophilia. We used data from the prospective Age-related DeVelopments ANd ComorbiditiEs in hemophilia (ADVANCE) study.

The consequences of unemployment on both living standard and psychological well-being can be great. Therefore, enhancing the understanding of how treatment and other risk factors may influence employment is important.

## 2 Methods

### 2.1 Data collection and study recruitment

The ADVANCE Working Group is a collaborative effort uniting 20 hemophilia centers across 15 European countries. To investigate the unique health challenges faced by aging individuals with hemophilia, the group’s initial research was a cross-sectional study of hematuria and hypertension in hemophilia A and B in people with hemophilia aged 40 years and older [13]. Building on this previously compiled data set, the ADVANCE Working Group launched a broader investigation. The new study, the ADVANCE study, employed a prospective, noninterventional, multicenter, and observational study design. To investigate aging in the hemophilia population, a consensus decision by the ADVANCE steering committee determined an age cutoff of 40 years or older. This decision considered both established knowledge and the goal of capturing arterial events.

The study was introduced to potential participants during their annual comprehensive clinic evaluation at their respective hemophilia treatment centers. A comprehensive electronic case report form (eCRF) was used, and the baseline eCRF included items related to patient characteristics, demographic information, past and current treatment regimens, and medical history with a lifelong history of comorbidities. The eCRF recorded whether a psychiatric disorder was present according to definitions established by the World Health Organization and the International Classification of Disease. The specific psychiatric diagnosis was not available in the research dataset.

After completing an informed consent form, study participants were scheduled for annual follow-up visits within a 10-year study. For the annual follow-ups, the eCRF was based on a “tick-box” approach and included variables whose data could be collected from the medical records; however, the form did not include items related to physiological and metabolic variables (eg, weight, blood pressure, and lipid profile, among others), and the follow-up questionnaire specifically asked for any changes since the past assessment.

The ADVANCE study recruited 785 participants aged 40 years or older with hemophilia A or B by November 2018. Participants self-reported employment as full time, part time, early retirement, retired, unemployed disabled, or unemployed not disabled. Respective national ethical committees or institutional review boards approved the study.

### 2.2 Statistical methods

Baseline data from the ADVANCE study were analyzed using descriptive statistics and logistic regression models. All analyses were performed using R (version 4.3.2; R Foundation for Statistical Computing) [14]. The tidyverse R package (version 2.0.0) [15] was used for data manipulation.

## 3 Results

### 3.1 Study population characteristics

Our study included 756 participants (aged 40-88 years; median, 53 years) with employment status data. The majority had hemophilia A (85%) and severe disease (56%). On-demand treatment was the most common (59%). Demographic and clinical characteristics of the people with hemophilia participating in the ADVANCE study are shown in Table 1. Ninety-seven percent of the participants identified as Caucasian. Due to the underrepresentation of other ethnicities, analysis of the relation between ethnicity and employment was not pursued in this study.

**Table 1:** Demographic and clinical characteristics of study participants.

Characteristic	Hemophilia A (*n* = 644)	Hemophilia B (*n* = 112)	Overall (*n* = 756)
Age (y)	53 (46-61)	54 (45-63)	53 (46-61)
Hemophilia severity			
Nonsevere	283 (44%)	50 (45%)	333 (44%)
Severe	361 (56%)	62 (55%)	423 (56%)
Treatment			
On-demand	383 (60%)	60 (54%)	443 (59%)
Prophylaxis	244 (38%)	50 (45%)	294 (39%)
Bypass therapy	7 (1.1%)	1 (0.9%)	8 (1.1%)
Smoker	144 (23%)	26 (24%)	170 (24%)
Psychiatric disorder	26 (4.1%)	3 (2.7%)	29 (3.9%)
Employment status			
Full-time work	328 (51%)	59 (53%)	387 (51%)
Part-time work	65 (10%)	5 (4.5%)	70 (9.3%)
Early retirement	36 (5.6%)	10 (8.9%)	46 (6.1%)
Retired	126 (20%)	24 (21%)	150 (20%)
Unemployed, disabled	55 (8.5%)	9 (8.0%)	64 (8.5%)
Unemployed, not disabled	34 (5.3%)	5 (4.5%)	39 (5.2%)
Ethnicity			
Caucasian	620 (96%)	112 (100%)	732 (97%)
Black	8 (1.2%)	0 (0%)	8 (1.1%)
Asian	6 (0.9%)	0 (0%)	6 (0.8%)
Other	10 (1.6%)	0 (0%)	10 (1.3%)
Chronic liver disease	150 (24%)	28 (25%)	178 (24%)
HIV-positive	121 (19%)	9 (8.3%)	130 (18%)
HCV-positive	163 (26%)	27 (24%)	190 (26%)
BMI			
Not overweight	278 (45%)	44 (40%)	322 (44%)
Overweight	344 (55%)	67 (60%)	411 (56%)
Inhibitor			
Never had inhibitor	572 (90%)	107 (96%)	679 (91%)
Previous inhibitor	38 (6.0%)	3 (2.7%)	41 (5.5%)
Current inhibitor	26 (4.1%)	1 (0.9%)	27 (3.6%)
Current target joint	124 (21%)	21 (20%)	145 (21%)

The numbers show median and IQR for continuous variables and number and percentage of category for categorical variables. The percent unemployed numbers in this table do not constitute the unemployment rate as not all categories should be included in the denominator when computing the unemployment rate. Retired people should be removed from the sample when computing the unemployment rate, for example. For each variable in the table, only nonmissing values are included in the counts. Thus, for variables that have missing values, the numbers do not add to the totals listed in the column header.

BMI, body mass index; HCV, hepatitis C virus.

**Figure 1:**
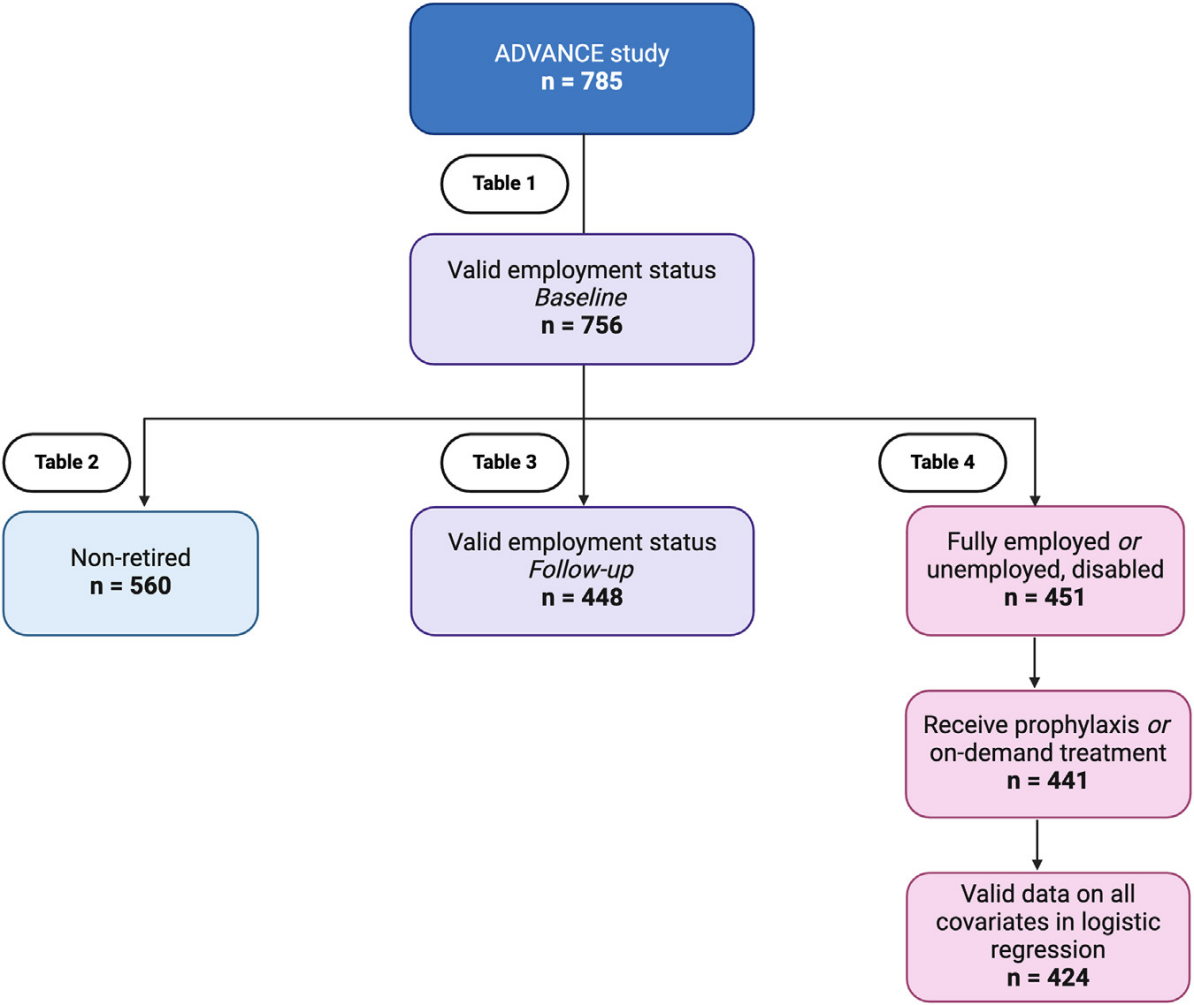
Sample selection criteria for the analyses included in this paper.

We performed various analyses of subsamples of the group with valid employment status data, such as those who are not retired, those with available follow-up data, and those who were included in the regression analysis (Figure 1).

### 3.2 Unemployment among people with hemophilia vs nondisabled general population

In the current study, people with hemophilia had higher unemployment rates than the general nondisabled population across most countries (Table 2). Disability was the primary reason for unemployment for people with hemophilia. The overall unemployment rate in the study was 18.4%, of which 11.4% was due to disability, compared with the 7.4% weighted average for the general population.

**Table 2:** Unemployment by country compared with OECD unemployment rates.

Country	OECD	ADVANCE				Weight in sample
	Total	Not disabled	Disabled	Total	*n*	
Austria	5.7	3.7	0.0	3.7	27	4.8%
Belgium	6.9	12.0	12.0	24.0	25	4.5%
France	9.7	15.6	18.8	34.4	32	5.7%
Germany	4.6	5.2	5.2	10.3	58	10.4%
Greece	21.4	0.0	0.0	0.0	4	0.7%
Israel	5.6	0.0	5.6	5.6	18	3.2%
Italy	11.5	7.1	1.4	8.6	70	12.5%
Netherlands	7.1	8.3	13.9	22.2	72	12.9%
Norway	4.0	9.6	19.2	28.8	52	9.3%
Poland	6.3	0.0	41.7	41.7	12	2.1%
Slovenia	6.8	0.0	14.3	14.3	7	1.3%
Spain	21.6	10.0	15.0	25.0	20	3.6%
Sweden	7.8	3.3	3.3	6.7	30	5.4%
Switzerland	4.8	7.7	34.6	42.3	26	4.6%
United Kingdom	5.5	5.6	10.3	15.9	107	19.1%
Total	7.4	7.0	11.4	18.4	560	100.0%

Countries have been weighted according to the number of patients from each country in the sample when computing the total. These weights were also used to construct an average for the general population. The OECD average was computed by taking the weighted average of unemployment rates according to the year of the first visit for the patients.

ADVANCE, Age-related DeVelopments ANd ComorbiditiEs in hemophilia; OECD, Organisation for Economic Co-operation and Development.

In contrast to the OECD definition, which excludes those unable to work due to disability, our study included all unemployed individuals, regardless of disability status when computing proportions. People who had retired were not included in the rates. Unemployment figures for the general population were sourced from OECD statistics [16].

### 3.3 Employment status over time

In this section, we included only patients with valid employment status at follow-up (*n* = 448). Longitudinal data showed limited changes in employment status between the baseline and the last follow-up (Table 3). The first column shows the baseline employment status, and the following columns show the status at the last follow-up. Panel A shows the actual number of patients. Sum totals for each row (baseline) and column (follow-up) have been computed. For example, at baseline 227 patients were in full-time work. At follow-up, 186 of these remained in full-time work. Panel B shows the numbers converted to percentages. For each row, the diagonal holds the highest percentage, showing that patients tend to remain in the employment category recorded at the first visit. Notably, few people with hemophilia transitioned from full-time work to unemployed (disabled) and vice versa, limiting insights from follow-up analysis. Therefore, the regression analysis used only initial visit data.

**Table 3:** Employment status at baseline and follow-up.

Employment status	Full-time work	Part-time work	Early retirement	Retired	Unemployed, disabled	Unemployed, not disabled	Total
Panel A: distribution of patients going from baseline category to follow-up categories							
Full-time work	**186**	16	2	16	1	6	227
Part-time work	9	**16**	2	5	4	0	36
Early retirement	3	0	**11**	9	2	1	26
Retired	6	0	3	**78**	2	1	90
Unemployed, disabled	2	3	0	8	**26**	3	42
Unemployed, not disabled	4	1	0	6	4	**12**	27
Total	210	36	18	122	39	23	448
Panel B: distribution of patients going from baseline category to follow-up categories							
Full-time work	**82%**	7%	1%	7%	0%	3%	100%
Part-time work	25%	**44%**	6%	14%	11%	0%	100%
Early retirement	12%	0%	**42%**	35%	8%	4%	100%
Retired	7%	0%	3%	**87%**	2%	1%	100%
Unemployed, disabled	5%	7%	0%	19%	**62%**	7%	100%
Unemployed, not disabled	15%	4%	0%	22%	15%	**44%**	100%

The first column shows the baseline status, and the subsequent columns show the status at the last follow-up. The diagonal bolded values denote patients who remained in their baseline category at follow-up.

### 3.4 Severity, treatment, and employment

For a clearer analysis, in the remainder of the results, we limited the sample to people with hemophilia who received either prophylaxis or on-demand treatment and who were either unemployed due to disability or fully employed. While it is likely that some people with hemophilia sought part-time work because full-time work would have been too taxing because of hemophilia, there could be other explanations as well, such as preferring to work less. Therefore, the cleanest comparison was between full-time work and unemployment due to disability.

**Figure 2:**
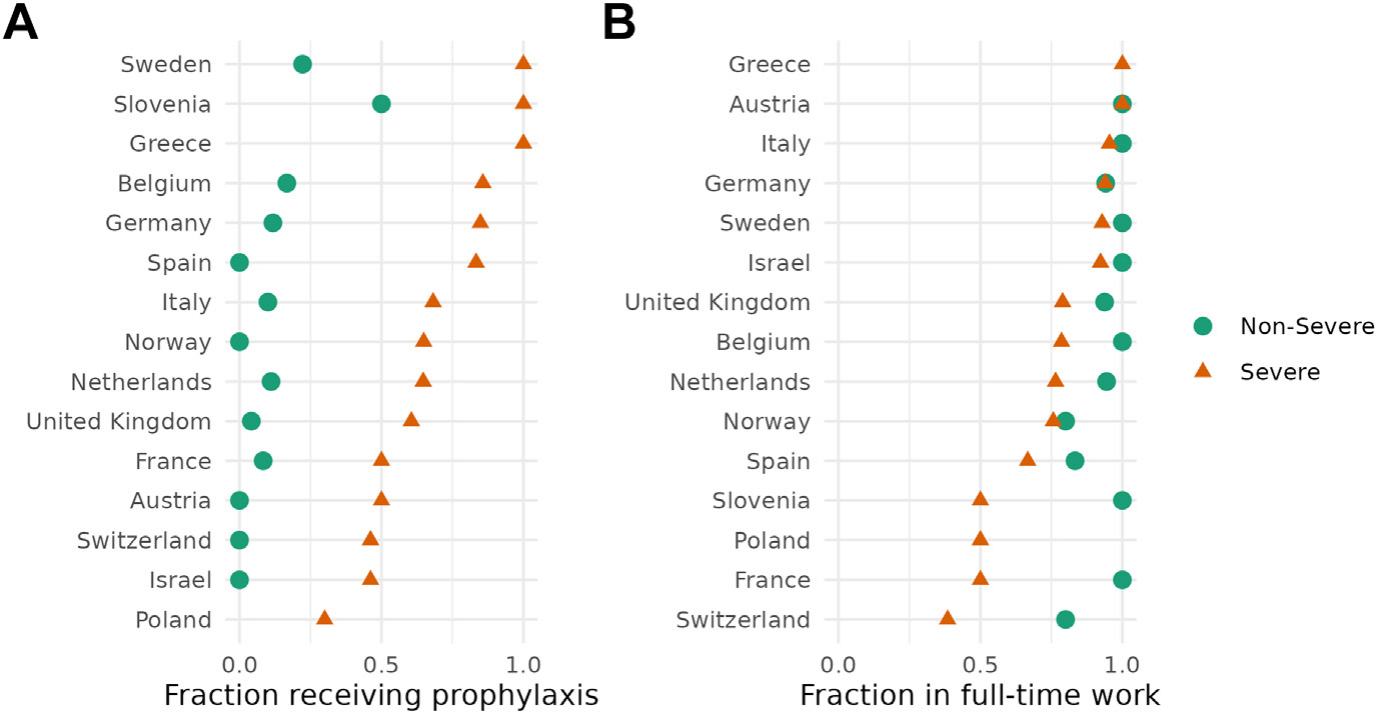
Fractions of patients (A) receiving prophylaxis and (B) in full-time work by country.

**Figure 3:**
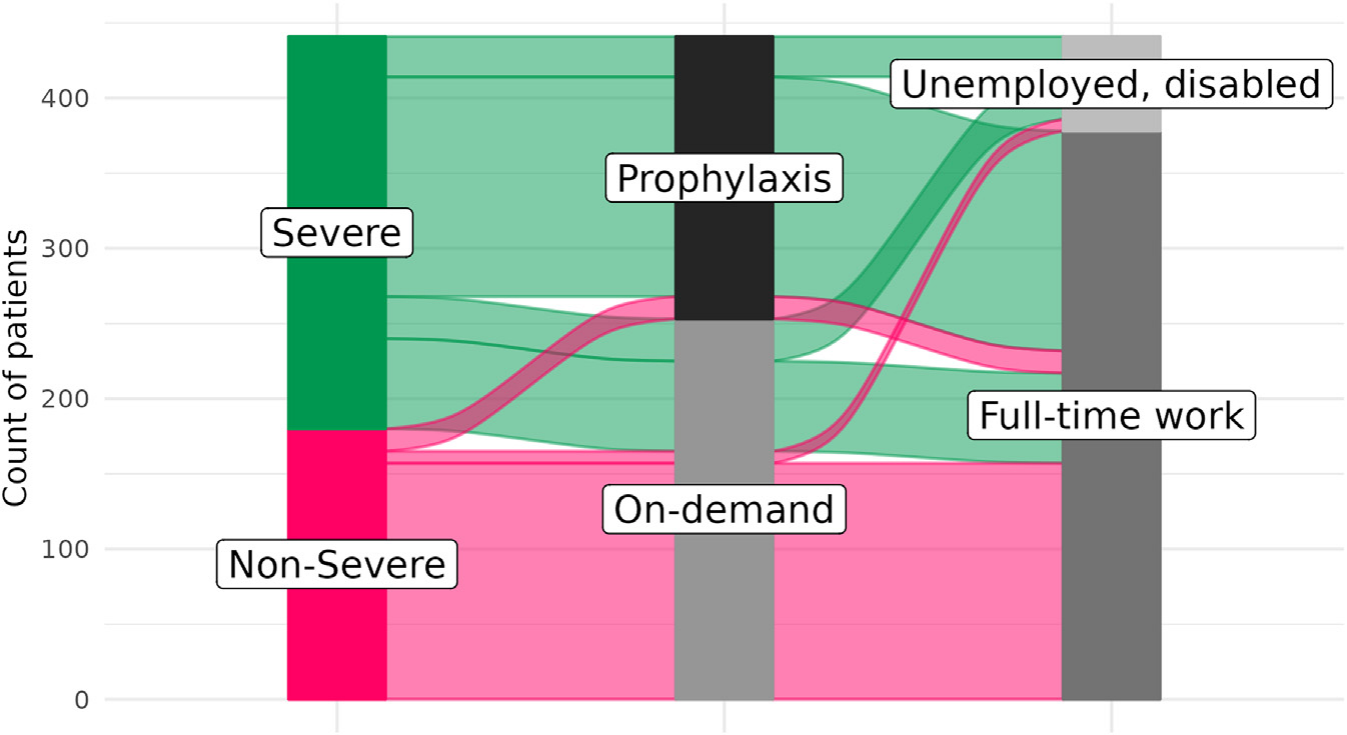
Flow diagram of severity, treatment, and employment status for patients in the sample.

People with severe hemophilia were more likely to receive prophylaxis (Figure 2A) and less likely to be fully employed (Figure 2B). Figure 3 further illustrates the relation between severity, treatment, and employment status in the cross-sectional sample recorded at the first visit. The majority of those with severe hemophilia received prophylactic treatment, but a substantial minority received on-demand treatment. Among those with nonsevere hemophilia, very few received prophylaxis. Unsurprisingly, most patients who were unemployed due to disability suffered from severe hemophilia.

### 3.5 Factors associated with unemployment

Multivariable logistic regression models were used to examine the associations between employment status, treatment, and other covariates that previous literature has shown to be related to unemployment. Our regression analyses focused on full-time employment vs unemployment due to disability to isolate the impact of hemophilia on work status and to avoid the influence of factors related to choosing part-time work.

Table 4 shows results from univariable and multivariable logistic regression models (*n* = 424), where the dependent variable is employment status, with levels unemployed due to disability and fully employed (baseline). People with hemophilia on prophylaxis had significantly lower odds of unemployment due to disability (odds ratio [OR], 0.44; *P* = .01). In contrast, severe hemophilia (OR, 10.81; *P* < .01), older age (OR, 1.07; *P* < .01), current smoker (OR, 2.53; *P* < .01), and psychiatric disorder (OR, 4.18; *P* = .02) were significantly associated with higher odds of being unemployed due to disability. The presence of chronic liver disease and a current target joint was associated with unemployment due to disability in univariable analysis, but not in multivariable logistic regressions after adjusting for severity. There was no association with type of hemophilia, low or high body mass index, concomitant infectious diseases (HIV and hepatitis), presence of inhibitor (previous or current), or other comorbid conditions.

**Table 4:** Logistic regression of employment status.

Covariate	Levels	Full-time work (*n* = 362)	Unemployed, disabled (*n* = 62)	Odds ratio (univariable)^a^	Odds ratio (multivariable)^a^
Treatment	On-demand	217 (85.8)	36 (14.2)	-	-
	Prophylaxis	161 (85.6)	27 (14.4)	1.01 (0.59-1.73; *P* = .97)	0.44 (0.23-0.83; *P* = .01)
Age (y)	Mean (SD)	49.8 (7.0)	52.2 (8.2)	1.05 (1.01-1.08; *P* = .01)	1.07 (1.03-1.11; *P* < .01)
Hemophilia severity	Nonsevere	172 (95.6)	8 (4.4)	-	-
	Severe	206 (78.9)	55 (21.1)	5.74 (2.81-13.34; *P* < .01)	10.81 (4.77-27.35; *P* < .01)
Smoker	No	282 (88.1)	38 (11.9)	-	-
	Yes	82 (77.4)	24 (22.6)	2.17 (1.22-3.81; *P* = .01)	2.53 (1.35-4.71; *P* < .01)
Psychiatric disease	No	367 (86.4)	58 (13.6)	-	-
	Yes	9 (64.3)	5 (35.7)	3.52 (1.05-10.55; *P* = .03)	4.18 (1.13-14.21; *P* = .02)

The covariates include 4 binary variables (treatment, severity, smoker, and psychiatric disease) and 1 continuous variable (age). The second column shows the levels of the binary variables. For the binary variables, the third and fourth columns show the number of patients who are employed and unemployed for each level of the variable. The numbers in parentheses convert the counts to a percentage. For the continuous variable, these columns show the mean and the SD in parentheses.

a The ranges represent the 95% confidence interval.

## 4 Discussion

Our study highlights the significant challenges faced by people with hemophilia in maintaining employment. People with hemophilia were much less likely to be employed than the nondisabled population. This disparity was largely driven by disability, emphasizing the often debilitating consequences of the disease and its associated comorbidities. We found that people with hemophilia who received prophylactic treatment were less likely to experience unemployment due to disability. The reduction in pain, bleeding episodes, and improved joint health associated with prophylaxis likely allowed people with hemophilia to engage in daily activity and pursue full employment.

Our main interest in this study was what impeded people with hemophilia from employment. Given the debilitating nature of severe hemophilia and its frequent comorbidities, studying these challenges is important as unemployment often worsens financial stability and mental well-being. Moreover, the psychological toll of unemployment can be severe, and people with hemophilia are already at a higher risk of depression [17,18].

There are several reasons for different rates of unemployment across European countries. Differences in the social safety nets, such as unemployment insurance programs play a role. For instance, generous unemployment benefits reduce the incentive to seek work, and a weak labor market may induce older workers to retire and workers with infirmities to opt for disability benefits. To assess this issue, we compared unemployment data from the baseline in the ADVANCE study with OECD unemployment statistics for each country and year. We found that people with hemophilia in the ADVANCE study were much less likely to be employed than people with no disability. Total unemployment (including both disabled and nondisabled) among people with hemophilia was approximately 2.5 times higher than country- and year-matched results for nondisabled persons. We are aware that people who are unemployed due to disability are not included in the OECD unemployment rate. Our intention was, however, to highlight that in total the unemployment rate among people with hemophilia not due to disability is comparable with the weighted OECD rate. When including unemployment due to disability, the fraction of people with hemophilia who were not employed far exceeded the OECD unemployment rate, emphasizing the fact that many people with hemophilia are excluded from the labor market.

At the last follow-up visit, only 2 persons (5%) had gone back to full-time work and 3 more (7%) had gone back to part-time work after being unemployed due to disability. The paucity of movement between the employment categories highlights the importance of preventing unemployment due to disability in the first place as the majority of people with hemophilia who become unemployed remain so. With such a low re-employment rate, many people with hemophilia are missing opportunities for contributing their skills to the workforce.

### 4.1 Key findings

In this section, we discuss the implications based on the logistic regression models, with a particular emphasis on the link between hemophilia treatment and employment.

#### 4.1.1 The potential benefit of prophylaxis

The main finding in this study was the negative association between prophylaxis and unemployment due to disability in the multiple logistic regression model. The multiple regression OR for prophylactic treatment was 0.44, indicating that prophylaxis was associated with a significantly lower probability of being unemployed due to disability. We did not observe a significant association between treatment and employment in a univariable context. The explanation for this is straightforward and highlights the importance of adjusting for the severity of hemophilia in multiple logistic regression. Most people with nonsevere hemophilia did not receive prophylaxis, and they were also more likely to be employed full time. Thus, our analysis suggests that administering prophylactic treatment to people with hemophilia, compared with nonregular on-demand treatment, could help avoid unemployment due to disability.

#### 4.1.2 What determines type of treatment?

The type of treatment is determined by several factors In some countries, the choice is given. Sweden, for example, has a long history of pioneering prophylaxis in hemophilia [19], a practice later adopted by other Nordic countries. Also, in Nordic countries, prophylaxis is increasingly available for those with moderate hemophilia with a bleeding phenotype [20]. In other countries, the choice is left to the discretion of the attending physician. However, the latest guidelines from the World Federation of Hemophilia strongly advocate for prophylaxis with trough levels of 3%-5% as the recommended primary treatment for hemophilia [21]. This has also been the target threshold for the people with hemophilia receiving prophylaxis in the ADVANCE study. This is recommended even in countries with limited healthcare resources, as the primary benefit of prophylaxis lies in its ability to enhance quality of life, not only cost-effectiveness. Despite its benefits and the recommendations, implementation of prophylaxis still faces problems in certain countries owing to medical, psychosocial, and economic considerations [22,23]. Even if there are drawbacks to prophylaxis, one must consider the benefits if treatment could imply employment and thus alleviate some of the hardships that people with hemophilia face.

#### 4.1.3 Other covariates associated with employment status.

There is a positive association between age and unemployment due to disability, meaning that the older people with hemophilia are less likely to be fully employed. This relation is distinct from other age-related reasons for not working full time, such as retirement or early retirement, as those patients have been removed from the sample in this regression model. One explanation is that hemophilia is a disease that gradually takes its toll on patients’ health, thereby increasing the likelihood of disability with age.

Severe hemophilia is also strongly positively associated with unemployment due to disability. Previous research has documented the higher unemployment rate among those with severe hemophilia [24,25], demonstrating the ongoing challenges people with hemophilia face compared with the rest of the population. Interestingly, the regression coefficient on severe disease almost doubles from the univariable (5.74) to the multivariable (10.81) regression. It was the adjustment for treatment that contributed most to the increase in the odds for severity.

We found that smoking was positively associated with unemployment. Being a former smoker had no effect, so we collapsed smoking to a binary variable with levels current smoker and not current smoker. The finding that unemployment tends to be higher among smokers has been found in several studies of the general population [[26], [27], [28]].

Concomitant psychiatric disorder was associated with higher odds of being unemployed due to disability. While only 3% of our cohort had concomitant psychiatric disorder, we included this as an independent variable in our analysis due to known associations between hemophilia and mental health diseases, such as depression and anxiety [17,18,29,30]. We acknowledge the possibility of reverse causation, however, as unemployment can have a significant impact on a person’s mental health and increase the risk of developing psychiatric disorders.

#### 4.1.4 Covariates considered but not included in the final model

Hemophilic arthropathy, a consequence of recurrent joint bleeds, is a common condition among people with hemophilia. In our analyses, we found that people with hemophilia with current target joint was associated with unemployment due to disability in univariable, but not in multivariable analysis after adjusting for hemophilia severity. Similarly, after adjusting for severity, chronic liver disease and infectious diseases were no longer significant predictors.

We have not considered covariates with more than 10% missing observations. This implies that we have not included serious nonjoint bleeds in the final multivariable logistic regression model.

### 4.2 Implications for future research

While prophylaxis is more costly than on-demand treatment [31] and can be burdensome, our results suggest potential long-term economic benefits.

An interesting topic for future research would be to assess the cost of treatment vs the cost of unemployment, updating existing studies [32,33] with current European estimates. The cost of unemployment for a society is not confined to a person’s lack of contribution to the gross domestic product. Several countries in our study have an extensive welfare state, which means that the unemployed receive substantial benefits. The costs also include reduced quality of life for many who are involuntarily unemployed. Such unemployment can cause psychosocial stress, which can adversely impact mental health and lead to anxiety, depression, and reduced self-esteem, among other harmful symptoms. A study which attempts to include all costs may find that prophylactic treatment is the cheaper option for society in the long run, despite its higher immediate cost.

### 4.3 Strengths and limitations of this study

The ADVANCE study is one of the most comprehensive multicountry studies conducted on people with hemophilia, including information on a wide range of demographic and clinical variables. This is among the greatest strengths of the study, contributing to its statistical power and suitability for treatment recommendations. Our study, however, also has several limitations.

The study design does not account for selection bias with respect to treatment. From clinical experience, we know that prophylaxis often requires a high level of commitment from patients, which might be linked to stronger motivation to seek employment. This means we cannot rule out the possibility that there exists a confounding variable that affects both employment and choice of treatment. Consequently, it is possible that those receiving prophylaxis are systematically different from those opting for on-demand treatment with respect to their motivation to seek employment. The best way to avoid selection bias is to use randomization, but this option has not been available for this study.

It is also possible that results are biased due to omitted variables, such as lack of data on job type and duration. It is more difficult for people with hemophilia to perform heavy manual labor than office work. Thus, if a participant used to be a blue-collar worker, then arduous work may have taken a toll on the body, and ensuing unemployment due to disability could be more likely. White-collar work, in contrast, may be more conducive to remaining in full employment for people with hemophilia. Moreover, we do not have data on the duration of unemployment or whether persons have ever been employed.

In addition, since the baseline data from the ADVANCE study is cross-sectional, we cannot draw causal conclusions about the relation between unemployment due to disability and covariates analyzed in the study.

While missing data may prevent a complete analysis of the relation between treatment, work, and unemployment, this study strongly suggests a potential benefit of prophylaxis in improving the ability to obtain and maintain employment.

## 5 Conclusion

This study has detailed the elevated burden of unemployment among people with hemophilia and identified factors associated with unemployment. We found a negative association between prophylactic treatment and disability and detailed plausible explanations for this negative association. Based on our findings, we continue to recommend prophylaxis over on-demand treatment, and we think it compares favorably in terms of cost-effectiveness when a wider definition of cost is considered.

## Appendices

Members involved in Age-related DeVelopments ANd ComorbiditiEs in hemophilia at study initiation:

• Ingrid Pabinger, Vienna, Austria
• Cedric Hermans, Brussels, Belgium
• Roseline d’Oiron, Paris, France
• Robert Klamroth, Berlin, Germany
• Johannes Oldenburg, Bonn, Germany
• Natascha Marquardt, Bonn, Germany
• Peter Staritz, Heidelberg, Germany
• Olga Katsarou, Athens, Greece
• Uri Martinowitz, Tel Aviv, Israel
• Aharon Lubetsky, Tel Aviv, Israel
• Gili Kenet, Tel Aviv, Israel
• Annarita Tagliaferri, Parma, Italy
• Maria Elisa Mancuso, Milan, Italy
• Roger Schutgens, Utrecht, The Netherlands
• Pål André Holme, Oslo, Norway
• Jerzy Windyga, Warsaw, Poland
• Irena Zupan, Ljubljana, Slovenia
• Victor Jimenez Yuste, Seville, Spain
• Ramiro Nunez, Seville, Spain
• Philippe de Moerloose, Geneva, Switzerland
• Erik Berntorp, Malmö, Sweden
• Jan Astermark, Malmö, Sweden
• Campbell Tait, Glasgow, United Kingdom
• Gerry Dolan, London, United Kingdom
